# Genetic analysis using targeted next-generation sequencing of sporadic Chinese patients with idiopathic dilated cardiomyopathy

**DOI:** 10.1186/s12967-021-02832-3

**Published:** 2021-05-03

**Authors:** Mingmin Li, Shuang Xia, Lan Xu, Hong Tan, Junqing Yang, Zejia Wu, Xuyu He, Liwen Li

**Affiliations:** Department of Cardiology, Guangdong Cardiovascular Institute, Guangdong Provincial Key Laboratory of Coronary Heart Disease Prevention, Guangdong Provincial People’s Hospital, Guangdong Academy of Medical Sciences, Guangzhou, China

**Keywords:** Idiopathic dilated cardiomyopathy, *TTN*, *TNNT2*, *RBM20*, *FLNC*

## Abstract

**Background:**

Inherited dilated cardiomyopathy (DCM) contributes to approximately 25% of idiopathic DCM cases, and the proportion is even higher in familial DCM patients. Most studies have focused on familial DCM, whereas the genetic profile of sporadic DCM in Chinese patients remains unknown.

**Methods:**

Between June 2018 and September 2019, 24 patients diagnosed with idiopathic DCM without a family history were included in the present study. All patients underwent genetic screening for 80 DCM-related genes using targeted next-generation sequencing.

**Results:**

By in silico analysis, 10 of 99 detected variants were considered pathogenic or likely-pathogenic, including seven *TTN* truncating variants (TTNtv), one in-frame deletion in *TNNT2*, one missense mutation in *RBM20,* and one frameshift deletion variant in *FLNC*. Of these variants, eight are reported for the first time.

**Conclusions:**

Using targeted next-generation sequencing, potential genetic causes of idiopathic DCM were identified. Sarcomere mutations remained the most common genetic cause of inherited DCM in this cohort of sporadic Chinese DCM.

**Supplementary Information:**

The online version contains supplementary material available at 10.1186/s12967-021-02832-3.

## Background

Dilated cardiomyopathy (DCM) describes a group of myocardial disorders that lead to ventricular enlargement and compromised contraction. The reported incidence of DCM in China was 190 of every million in a stratified sampling investigation in 2002, and the 5-year mortality was ~ 50% in another report in 2014, respectively [1]. The aetiology underlying DCM is diverse and includes well-defined causes (for example, ischaemic injury) and unrecognised risk factors; hereafter, the latter condition has been referred to as idiopathic DCM. Rapid advancement in genetic technology led to the discovery of genetic mutations as direct causes of DCM 2 decades ago. Furthermore, genetic mutations were identified in 8–25% patients with idiopathic DCM [2]. Compared to sporadic cases, patients with a family history of DCM are more likely to have a genetic aetiology. Unlike the condition in hypertrophic cardiomyopathy, the genetic aetiology of DCM is highly heterogeneous. Thanks to high-throughput sequencing technologies, more than 50 DCM-related genes have been identified [3, 4]. The most frequent transmission mode is autosomal dominant, followed by autosomal recessive, X-linked, and mitochondrial inheritance. Most of the genes encode components of the sarcomere, cytoskeleton, or nuclear lamina. Nevertheless, few genetic variants have been proven to be disease-causing by robust experimental data [5]. Understanding the genetic background can offer new insights regarding the pathogenesis for the development of precision medicine of DCM. Most previous studies have focused on genetic screening of familial DCM. Based on these studies, familial and/or younger DCM patients are recommended for regular genetic screening at present. Otherwise, the genetic profile of sporadic DCM remains to be clarified. The current study aimed to discover the genetic background of sporadic idiopathic DCM in a group of Chinese patients.

## Methods

### Inclusion and exclusion criteria

This study included patients diagnosed with idiopathic DCM between June 2018 and September 2019. The diagnostic criteria for idiopathic DCM were consistent with the guidelines described by the AHA Scientific Statement and position statement of the ESC working group [3, 6].

The exclusion criteria included peripartum cardiomyopathy and secondary dilated cardiomyopathies caused by ischaemic heart diseases, hypertension, valvular diseases, endocrine disorders (such as diabetic cardiomyopathy and hyperthyroid cardiomyopathy), inflammation, toxicity, and stress. In a recently published study, Ware and his colleagues found a similar genetic profile of well-characterised DCM-causing genes between DCM and alcoholic cardiomyopathy [7]. Hence, patients who consumed excess alcohol were not excluded from the study.

### Subjects and clinical evaluations

A total of 24 unrelated patients were included in the cardiology department of Guangdong Provincial People’s Hospital during the study period. Patients’ clinical data at the time of enrolment, including medical history, family history, physical examination, blood tests, 12-lead echocardiogram (ECG), transthoracic echocardiography, and/or cardiac magnetic resonance imaging were collected.

This study was approved by the Guangdong Provincial People’s Hospital ethics committee and met the Tenets of the Declaration of Helsinki. All included patients provided informed consent.

### DNA sample collection and targeted next-generation sequencing (NGS)

Peripheral venous blood was collected from the included patients and their relatives (if available) for genomic DNA isolation. The DNA was extracted using the Magnetic Universal Genomic DNA Kit (TIANGEN) according to the manufacturer’s protocol. To cover the coding sequence region and flanking sequences (adjacent intronic regions essential for splicing) of DCM-related genes, a customised panel of 179 genes associated with inherited heart diseases (including 80 genes related to inherited DCM, see Additional file 1) was used for the target sequence library. Genomic DNA was fragmented between 180–280 bp and 500 ng of each prepared library was recommended for hybrid capture. Amplicon libraries and hybrid capture were prepared using IDT xGen Exome Research Panel v1.0 kits, which was performed in accordance with the manufacturer’s instructions (Integrated DNA Technologies, Inc. Iowa, USA). The library was quantified with a Qubit 2.0 fluorometer (Invitrogen; Thermo Fisher Scientific). NGS was performed using Illumina NovaSeq 6000 System (Illumina, USA) by Novocardio Genetic Technology Co., Ltd. (Beijing, China). The sequencing data were collected and filtered for quality (see details in Additional file 2: Figure S1). The resultant data were subsequently aligned to the human reference genome assembly (Feb. 2009, GRCh37/hg19) using Burrow-Wheeler Aligner. Single nucleotide variants and insertions and deletions (indels) were detected using SAMtools [8] and an in-house filter pipeline (Additional file 2: Figure S1). At least 99% of the targeted bases were sequenced to a read depth of more than 20 times.

### Variant annotation and classification

Variants were identified using the Genome Analysis Toolkit, CNVkit, and annotated with ANNOVAR (http://wannovar.wglab.org/). All variants were confirmed by traditional Sanger sequencing. Common genetic variants or single nucleotide polymorphisms [with a minor allelic frequency (MAF) ≥ 1%] were excluded by referring to genetic databases, including dbSNP database (https://www.ncbi.nlm.nih.gov/snp/), 1000 Genomes Project (http://www.internationalgenome.org/categor y/dbsnp/), and Exome Aggregation Consortium (ExAC) database (details are provided in the Additional file 3). Variants leading to amino acid changes were predicted using the bioinformatics programmes SIFT (http://sift.jcvi.org/), Polyphen2 (http://genetics.bwh.harvard.edu/pph2/), and MutationTaster (http://www.mutationtaster.org/). The pathogenicity of a certain variant was defined according to the guidelines of the American College of Medical Genetics and Genomics (ACMG) and Association of Molecular Pathology (AMP) as follows: pathogenic (P), likely-pathogenic (LP), variant of uncertain significance (VUS), likely benign (LB), and benign (B) [9].

## Results

### Clinical characteristics

Of the 24 unrelated patients included in this study, there were 19 males and 5 females; the mean age at onset of DCM was 40 years. None of these patients had a family history of cardiomyopathy or sudden death. No extracardiac features were identified. Up to 25% of the cases had traditional cardiovascular risk factors, such as smoking and hypertension. Of these cases, 87.5% had class III or IV heart failure function. The mean left ventricular ejection fraction (LVEF) at enrolment was 27.0 ± 9.9% and mean left ventricular end diastolic dimension was 70.1 ± 7.8 mm. The median serum N-terminal B-type natriuretic peptide level was 2057.5 pg/mL and it ranged from 698 to 18381 pg/mL. With regard to arrhythmias, 20% patients had either ventricular tachycardia [8%, with implantable cardioverter-defibrillator (ICD)], atrial fibrillation (8%), or bundle branch block (4%). Most patients were treated with guideline-directed medical therapy. The patient clinical presentations are summarised in Table 1.

**Table 1:** Baseline clinical characteristics of enrolled dilated cardiomyopathy cases

Characteristics	(n = 24)
Age at onset, years	39.8 ± 12.3
Female gender, n (%)	5 (21%)
Hypertension, n (%)	6 (25%)
Diabetes mellitus, n (%)	2 (8%)
Smoking	6 (25%)
Alcohol	4 (17%)
Cholesterol, mmol/L	4.7 ± 1.6
LDL-c, mmol/L	3.2 ± 1.1
NT-proBNP, pg/mL	2057.5 (698,18,381)
NYHA III or IV, n(%)	21 (87.5%)
ICD, n (%)	2 (8%)
Atrial fibrillation	2 (8%)
Complete RBBB/LBBB	1 (4%)
LVEF, %	27.0 ± 9.9
LVEDD, mm	70.1 ± 7.8
LA diameter, mm	49.4 ± 9.6
ACEI/ARB/ARNI, n (%)	18 (75%)
Beta-blockers, n (%)	21 (87.5%)
Spironolactone, n (%)	22 (91.7%)
Diuretics, n (%)	21 (87.5%)

All variables are given as either number present (% of total number) or mean ± SD, except for NT-proBNP (N-terminal B-type natriuretic peptide), which is given as mean and range

NYHA, New York Heart Association functional classification; ICD, implantable cardioverter defibrillator; RBBB/LBBB, right bundle branch block/left bundle branch block; LVEDD, left ventricular end-diastolic dimension; LA, left atrium; ACEI/ARB/ARNI, angiotensin converting enzyme inhibitor/angiotensin receptor blocker/angiotensin receptor-neprilysin inhibitor

### Genetic variants

In total, 99 variants distributed across 27 DCM-related genes were identified. All variants either had an allele frequency < 1% in the 1000 genome project and ExAC projects, or could not be found in the local database. Based on in silico analysis and ACMG guidelines, 10 variants from 10 patients were considered likely-pathogenic or pathogenic. Other variants classified as VUS or benign were not included in further analysis (details are provided in the Additional file 4). The pathogenic variant was an in-frame deletion leading to an amino acid deletion in the coding sequence of *TNNT2*. The mutation types of other putatively likely-pathogenic variants were: three nonsense mutations and four frameshift small deletions located in *TTN*, one missense mutation in *RBM20,* and one frameshift small deletion variant in *FLNC*. All mutations were heterozygous. Mutations in *TTN* were the most common variants in this group of sporadic Chinese DCM patients. Detailed information on the putatively pathogenic/likely-pathogenic variants is summarised in Table 2.

**Table 2:** Pathogenic and likely-pathogenic variants

Patient ID	Gene	Chromosomal location (GRCh37)	Nucleotide change	Amino acid change	Ref sequence Protein region	Mutation type	Allele frequency	ACMG criteria/pathogenicity	References
1	*TTN*	Chr2:179,444,906–179,444,907	c.62184_62185delCT	p. Asp20728fs	NM_001256850.1: exon268, A-band	Frameshift (truncating)	**–**	PVS + PM2; LP	None
5	*TTN*	Chr2:179,486,443	c.40185delT	p.Asn13395fs*11	NM_001256850.1: exon195, I-band	Frameshift (truncating)	**–**	PVS + PM2; LP	None
7	*TTN*	Chr2:179,425,091	c.80845C > T	p.Arg26949*	NM_001256850.1: exon276, A-band	Nonsense (truncating)	8E-06	PVS + PM2; LP	rs748689777
9	*RBM20*	Chr10:112,572,067	c.1912C > T	p.Pro638Ser	NM_001134363 exon9	Missense	**–**	PM1 + PM2 + PM5 + PM6 + PP3; LP	None
10	*TTN*	Chr2:179,486,346	c.40282G > T	p.Glu13428*	NM_001256850.1: exon195; I-band	Nonsense (truncating)	**–**	PVS + PM2; LP	None
15	*TTN*	Chr2:179,470,153	c.48946C > T	p.Gln16316*	NM_001256850.1: exon229, A-band	Nonsense (truncating)	**–**	PVS + PM2; LP	None
18	*TTN*	Chr2:179,466,820	c.50255delG	p.Gly16752Ala fs*30	NM_001256850.1 exon234, A-band	Frameshift (truncating)	**–**	PVS + PM2; LP	None
22	*FLNC*	Chr7:128,483,501	c.2681delC	p.Lys895Arg fs*27	NM_001127487 exon18	Frameshift (truncating)	**–**	PVS + PM2; LP	None
23	*TNNT2*	Chr1:201,331,099–201,331,101	c.650_652delAGA	p.Lys217del	NM_000364 exon13	Deletion	**–**	PS3 + PM1 + PM2 + PM4; P	rs45578238
24	*TTN*	Chr2:179,425,848–179,425,851	c.80085_80088delTAGT	p.Glu26697His fs*9	NM_001256850.1 exon276, A-band	Frameshift (truncating)	**–**	PVS + PM2; LP	None

LP, likely pathogenic; P, pathogenic

In addition, Sanger sequencing for the detected mutations could be carried out for the relatives of three patients. For the patient with an *RBM20* missense mutation, both his parents showed wild-type alleles, indicating a de novo mutation. Patient No. 15, a carrier of a heterozygous nonsense mutation in *TTN*, has two daughters; the older daughter showed the same mutation as the index patient, and the other was wild-type. The patient with an in-frame deletion in *TNNT2* died during index hospitalisation; both his mother and uncle were heterozygous for the same *TNNT2* mutation and remained healthy at the time of enrolment.

## Discussion

Mutations in *TTN* were the most common variants in the current group of sporadic Chinese DCM patients. Together with an identified *TNNT2* variant, sarcomere mutations seemed to contribute most to the genetic cause of this idiopathic DCM cohort, which is consistent with previous reports [4, 10, 11]. However, mutations in *MYBPC3* and other sarcomere genes were not detected in this cohort.

### Variants in *TTN*

The *TTN* gene encodes the sarcomere protein titin, the third most abundant striated-muscle protein after myosin and actin. Two titin molecules with opposite polarity span each sarcomere, the contractile unit in the myocardium. Thereafter, normal expression of *TTN* is essential for sarcomere assembly and function as a contractile unit. Titin is a large human protein, composed of approximately 34,000 amino acids at full length. There are different isoforms of titin depending on alternative splicing, of which, N2BA and N2B isoforms are dominantly expressed in the adult myocardium. The N2BA isoform, which is longer and more compliant than N2B, is abundant in foetal and neonatal heart. During development, a smaller and stiffer N2B isoform is predominantly expressed. Hence, the ratio of titin isoforms plays a key role in regulating cardiac diastolic function [12].

*TTN* heterozygous mutations are reported to be major genetic causes of DCM [13, 14]. Herman and his team revealed that *TTN* truncating variants (TTNtv) are a common cause of DCM, with a reported incidence rate of approximately 25% and 18% in familial idiopathic DCM and sporadic cases, respectively [10]. Nevertheless, not all TTNtv genes are pathogenic. It has been reported that TTNtv occurs in ~ 2% of the general population [15]. DCM-associated mutations are usually enriched in the titin A-band, which is close to the carboxy-terminus of the protein and highly conserved across most isoforms (Additional file 5: Figure S3). In other words, the clinical significance of TTNtv is mainly predicted by exon usage and variant location. In addition, in vitro analysis demonstrated that the mutant truncated titin protein in iPS cell-derived cardiomyocytes resulted in sarcomere insufficiency and impaired cell signalling activation, which might be the underlying mechanism of DCM-associated TTNtv [16]. However, the exact mechanisms by which TTNtv leads to DCM remain unknown.

In our study, *TTN* truncating variants were detected in 7 of the 24 tested patients; these included four frameshift deletions and three nonsense mutations. Except for Arg26949* (rs748689777), six variants are reported for the first time. The Arg26949* variant has been reported previously in both DCM and peripartum cardiomyopathy [10, 17]. It’s located in the A-band region of titin, where most DCM-related *TTN* truncating mutations are clustered. Among the six novel mutations, four clustered in the A-band, and the other two variants were located in exon 195 of the I-band region. Except for in silico functional prediction, no functional analysis of these variants was available. According to the pathogenicity prediction model by Roberts et al. TTNtv affecting highly-expressed exons have a predicted 93% probability of pathogenicity in DCM patients [15]. Another study of several large DCM cohorts confirmed this prediction model for the pathogenicity of TTNtv in DCM patients [18]. Exon 195 was highly expressed in the I-band. Therefore, together with the in silico analysis, we assumed that all seven TTNtv genes were likely pathogenic.

It is noteworthy that there is an observed association between TTNtv location and patient phenotype in a small cohort. Patients with a carboxy-terminal TTNtv, which truncates principal isoforms, are likely to present with severely impaired left ventricular function and life-threatening ventricular arrythmias [15, 19]. However, in a larger prospective study that included 83 DCM patients carrying TTNtv [20], no significant relationship between TTNtv location and severity of the phenotype was observed. Furthermore, except for an earlier onset age, a higher proportion of positive family DCM history, a higher occurrence of ventricular tachycardia, and fewer conduction diseases for patients with TTNtv, significant differences were not observed in clinical features or the medium-term cardiac outcome including cardiovascular death, arrhythmic events, and heart failure between patients with and without TTNtv.

In this study, all seven TTNtv carriers presented with clinical symptoms of heart failure after 30 years of age, with mean onset age of 42 years around. The mean LVEF was 26.6 ± 6.8% and the mean LVEDD was 68.4 ± 2.7 mm. Out of seven TTNtv carriers, six had class III or IV heart failure function. No extracardiac features or ventricular arrhythmias were detected. With regard to arrhythmias, one patient had atrial fibrillation and another had complete right bundle branch block at enrolment. As the most frequent variants detected in this small cohort, there were no significant differences between TTNtv carriers and those without TTNtv regarding onset age, LVEF and LVEDD (see Additional file 6: Table S3). However, all TTNtv carriers were males and none of them had ventricular tachycardia with or without ICD implanted, which were inconsistent with previous reports [10, 20]. As a small cohort, the association between genetic variants and clinical features should not be overinterpreted. The patient with the Gln16316* mutation had two daughters; the older daughter carrying the same TTNtv mutation was 8 years old and no cardiac abnormality was detected. Hence, the penetrance of all detected TTNtv was 87.5%, which was lower than the 95% penetrance reported in previous reports [10, 15]. This might be due to a younger age at genetic screening in this study, four of whom completed the genetic test before the age of 40.

### *TNNT2* mutation

Since the first DCM pedigree with *TNNT2* mutation reported in 2000, the role of troponin complex mutations in the pathogenesis of inherited DCM has been extensively explored. The overall frequency of troponin complex mutations in familial DCM was reported to be 6% in one large genetic study of idiopathic DCM [21]. *TNNT2* encodes troponin T, a subunit of the troponin complex, which plays an important role in generating cardiac contractility. The pathogenic mutation K217del (also known as p.K210del) in this study resulted in deletion of one lysine in the troponin T protein, leading to significant protein function abnormality. It has previously been reported in different familial DCM pedigrees [21–24]. The mutation results in a lysine deletion localised in exon 13, a conserved and functionally important domain involved in interaction with other troponin subunits or tropomyosin [21]. Functional analysis has demonstrated that the mutant proteins reduce the Ca^2+^ sensitivity of cardiac muscle contraction, resulting in force generation deficiency by sarcomere [25]. Furthermore, introduction of the *TNNT2* K217del mutation in mouse led to induction of a DCM phenotype with reduced survival [26].

In most pedigrees, the mutant allele co-segregated well with the DCM phenotype and was believed to be a recurrent mutation observed in DCM patients across different races [21, 22, 27]. Although most K217del carriers presented with early onset (usually before age 30) and aggressive DCM or even a sudden death, a milder presentation occurred in a few patients who stayed alive until their 60 s and 70 s [22, 23]. In our study, the patient who had the K217 deletion in *TNNT2* presented with acute heart failure at 14 years of age, and it deteriorated rapidly after onset of cardiac shock. Over a month of advanced medical support including IABP and ventilation, he did not survive in the end. We collected his parents’ and uncle’s blood sample for genetic testing, which revealed that both the mother and uncle were carriers for the same mutation as in the proband. Further clinical evaluation was performed for both mutation carriers, but no cardiac abnormalities were detected. Hence, the genotype–phenotype relationship in inherited DCM is complicated, even in troponin T cardiomyopathy, which is usually expressed as an aggressive and early onset disease, accompanied by considerable mortality.

### *RBM20* variant

*RBM20* encodes an RNA binding motif protein 20, a member of the SR protein family (serine/arginine-rich protein), which is highly expressed in the heart and regulates alternative RNA splicing by processing pre-messenger RNA (Additional file 7: Figure S4). In fact, *RBM20* regulates splicing of a set of genes, including *TTN*, and these target proteins are necessary for normal cardiac structure and signal transduction [12]. According to published large cohort studies, *RBM20* mutations account for approximately 3% of all DCM cases [28, 29].

Brauch et al. first reported five unique *RBM20* mutations co-segregating with DCM across eight pedigrees. They found all five mutations clustered within exon 9, encoding an RS-rich domain (a major domain of RS protein, rich in arginine–serine repeats). The altered amino acid residues (residues 634, 636, 637, and 638) were highly conserved across different species and were demonstrated to be a mutation hotspot [28]. In addition, Filippello and his colleagues revealed that the amino acids 491–658 contributed to RBM20 nuclear localisation and normal function of alternative splicing [30]. An in vivo functional analysis with a missense mutation S635A in RBM20 revealed that the mutant RBM20 changed the titin isoform expression, leading to the production of an abnormal higher-molecular-weight titin isoform, thereby promoting cardiac diastolic function and fibrosis [31]. In our study, the P638S mutation was identified in one patient who presented with heart failure at the age of 17 years. Both parents were screened negative for this variant, indicating a de novo mutation. The altered residue occurred in the mutation hotspot for *RBM20*. Although this amino acid change is first reported herein, missense mutations at the same residue, such as P638L, has been reported previously [28]. As functional analysis has demonstrated the loss of function of mutant P638L [31], combined with ACMG criteria, P638S is assumed to be likely-pathogenic.

DCM patients carrying pathogenic *RBM20* mutations were reported to have an early onset of disease compared to those without a definite genetic cause [28]. In addition, male carriers tended to have an aggressive disease progression, and one-third of the affected cases died suddenly or experienced episodes of ventricular tachycardia [32]. However, over 30% of mutation carriers were asymptomatic upon genetic screening, and most of them were younger than their affected probands, suggesting an age-dependent penetrance of *RBM20* mutations. In this study, the patient with the P638S mutant presented with heart failure at his teenage years, which was consistent with previous reports [28, 32]. During the index hospitalisation, no ventricular arrhythmia was observed.

### *FLNC* mutation

*FLNC* encodes protein filamin C, which cross-links actin filaments and constitutes a widespread network in both cardiac and skeletal muscle cells (Additional file 8: Figure S5). In cardiac myocytes, filamin C protein is highly expressed and participates in mechanical and signal transduction between sarcomeres and plasmatic membranes [33]. *FLNC* mutations were first recognised as genetic defects causing skeletal myofibrillar myopathy. As ~ 30% of affected myofibrillar myopathy patients carrying *FLNC* mutations presented with both skeletal myopathies and unspecified forms of cardiomyopathy [34], screening for *FLNC* mutations has been included in the genetic diagnosis of inherited cardiomyopathies since 2012. Ortiz-Genga et al. screened 2877 patients diagnosed with different inherited cardiovascular diseases for *FLNC* mutations and identified 23 truncating mutations in 28 probands [35]. Of these, 20 cases were diagnosed with DCM, and seven were arrhythmogenic cardiomyopathies. The prevalence of *FLNC* mutations in DCM patients was 3.9%. None of these patients expressed skeletal myopathies. However, a specific phenotype of a combination of significant left ventricle systolic dysfunction, myocardial fibrosis predominantly affecting the left wall, and a high burden of ventricular arrhythmias (approximately 82%) was observed in most affected patients carrying *FLNC* truncating mutations. Such clinical features observed in *FLNC* truncating mutations were confirmed in another genetic study of 319 DCM families, which identified 13 *FLNC* truncation carriers, with 85% having either ventricular arrhythmias or sudden cardiac death [36]. In addition, a histological study detected cardiac interstitial fibrosis in the right ventricle and fibrofatty infiltration in the left ventricle, which mimicked arrhythmogenic right ventricular cardiomyopathy to some extent. Except for DCM, some *FLNC* mutations were also related to hypertrophic and restrictive cardiomyopathies [37, 38]. However, consistent with the case of myofibrillar myopathy, variants leading to hypertrophic cardiomyopathy (HCM) or restrictive cardiomyopathy (RCM) were mostly missense mutations. Immunohistochemical staining of myocardial tissue from patients carrying *FLNC* truncations revealed no abnormal cytoplasmic filamin C aggregates, which was previously described in myofibrillar myopathy as well as in HCM and RCM patients, but showed decreased levels of normal filamin C [35–39]. Hence, a haploinsufficiency hypothesis for the underlying mechanism of *FLNC* truncating mutations was proposed.

The *FLNC* mutation detected in this study was a novel truncating variant. The patient who carried this K895Rfs mutation was a female, presenting with ventricular tachycardia and aborted ventricular fibrillation at the age of 26 years. An ICD was implanted for secondary prevention during index hospitalisation. No skeletal myopathy was detected, which was consistent with other previously reported *FLNC* truncating mutation carriers.

The overall prevalence of putatively pathogenic/likely-pathogenic mutations in this sporadic DCM cohort was 10 out of 24, indicating a higher proportion of genetic causes in idiopathic DCM even without a definite family history. Of course, this prevalence is overestimated due to lack of robust evidence to confirm the causative relationship between detected variants and DCM phenotype. Accidently, three relatives of the index patients were mutation carriers, as detected by genetic screening, suggesting that periodic cardiac evaluation ahead of any incidence of cardiac dysfunction might be useful. To our knowledge, the correlation between conduction or ventricular arrhythmia and *LMNA* mutations is the only confirmed genotype/phenotype relationship for genetic DCM based on meta-analysis of multiple DCM genetic studies [11]. The heterogeneous clinical presentations in genetic DCM with the same mutant gene might be a result of modifying factors, such as modifier genes and epigenetic factors. Therefore, the effect of genetic testing on clinical feature prediction and therapeutic guidance is limited. In specific genotypes, such as *FLNC* mutations, an ICD for primary prevention should be considered earlier.

This study has several limitations. First, it is based on a small cohort; hence, the result should not be over-interpreted. In addition, data are limited for analysis of genotype–phenotype correlation or clinical features for a specific genotype. Second, except for in silico analysis, our study does not yet provide robust evidence to confirm the causation between genetic variants and DCM phenotypes. Furthermore, we have not performed variants detection in healthy Chinese individuals as control. Hence, for previously reported variants we listed the allele frequency by referring to the public online database, such as the ExAC and the dbSNP database (see details in Table 2) and used the information as control. However, in order to save the cost of the study, the newly identified variants have no such background population variation for control.

## Conclusions

In conclusion, in a small Chinese cohort of sporadic idiopathic DCM, we identified a high proportion of inherited DCM, with frequently detected sarcomere mutations, especially mutations in the titin gene. Therefore, given patients’ willingness, genetic screening should be considered in young patients with idiopathic DCM, even without a positive family history.

## Supplementary Information

**Additional file 1: Table S1.** List of DCM related genes sequenced in this study.

**Additional file 2: Figure S1.** Variants filtration pipeline

**Additional file 3: Figure S2.** Variants annotation workflow.

**Additional file 4: Table S2.** List of all detected variants of DCM related genes.

**Additional file 5: Figure S3.** Structure and domains of *TTN.*

**Additional file 6: Table S3.** Characteristics of DCM patients, according to the presence or absence of TTNtv.

**Additional file 7: Figure S4.** Structure and domains of *RBM20.*

**Additional file 8: Figure S5.** Structure and domains of *FLNC*.

## Data Availability

All data generated or analysed during this study are included in this published article and its Additional files.

## Abbreviations

ACMG: American college of medical genetics and genomics
AHA: American Heart Association
DCM: Dilated cardiomyopathy
ESC: The European Society of Cardiology
ExAC: Exome aggregation consortium database
HCM: Hypertrophic cardiomyopathy
ICD: Implantable cardioverter-defibrillator
LP: Likely pathogenic
MAF: Minor allele frequency
NGS: Next generation sequencing
P: Pathogenic
RCM: Restrictive cardiomyopathy
TTNtv: *TTN* Truncating variants
VUS: Uncertain significance variants
