# Supplementary material for: Genetic analysis using targeted next-generation sequencing of sporadic Chinese patients with idiopathic dilated cardiomyopathy
Source: J Transl Med. 2021 May 3;19:189. doi: 10.1186/s12967-021-02832-3 (PMC8091742; doi:10.1186/s12967-021-02832-3)
Supplement: Supplementary file 1 — Additional file 1: Table S1. List of DCM related genes sequenced in this study. [file 12967_2021_2832_MOESM1_ESM.docx]

**Table S1:** **list of DCM related genes sequenced in this study.**

| CMD1B | EYA4 | DNAJC19 | PSEN2 | SDHA | ALMS1 | CMD1K | DCD1 |
| --- | --- | --- | --- | --- | --- | --- | --- |
| SOD2 | ADRB1 | PGM1 | SPEG | TGFB3 | CMD1Q | CMD1H | PDPK1 |
| TNNI3K | DOLK | JUP | LMNA | TNNT2 | SCN5A | TTN | PLN |
| MYH7 | RBM20 | BAG3 | MYPN | MYBPC3 | SGCD | DES | VCL |
| CSRP3 | ACTN2 | LDB3 | TPM1 | MYH6 | NEBL | DMD | CHRM2 |
| SYNE1 | ACTC1 | TNNI3 | TAZ | GATA5 | GATA6 | NKX2-5 | CASQ2 |
| ACTA1 | MYL2 | OBSCN | ABCC9 | TMPO | PSEN1 | FKTN | TNNC1 |
| DSG2 | NEXN | CRYAB | LAMA4 | PRDM16 | RAF1 | GATAD1 | ANKRD1 |
| CTF1 | DSC2 | EMD | FHOD3 | FLT1 | GATA4 | ILK | ISL1 |
| LAMA2 | LAMP2 | PDLIM3 | PKP2 | DSP | SYNM | TXNRD2 | FLNC |
