# Supplementary figures and images for: Genetic analysis using targeted next-generation sequencing of sporadic Chinese patients with idiopathic dilated cardiomyopathy
Source: J Transl Med. 2021 May 3;19:189. doi: 10.1186/s12967-021-02832-3 (PMC8091742; doi:10.1186/s12967-021-02832-3)

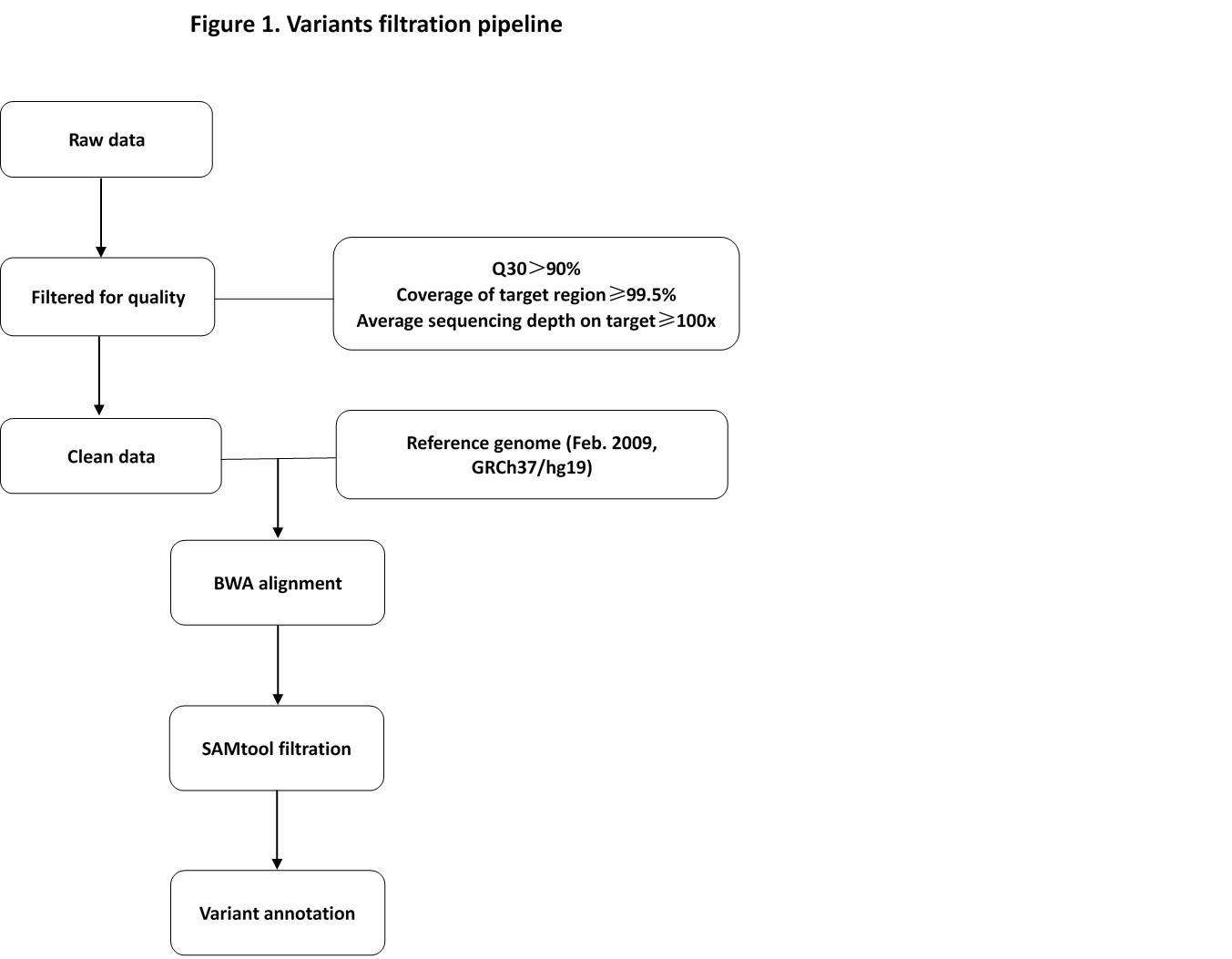

Supplement: Supplementary file 2 — Additional file 2: Figure S1. Variants filtration pipeline [file 12967_2021_2832_MOESM2_ESM.jpg]

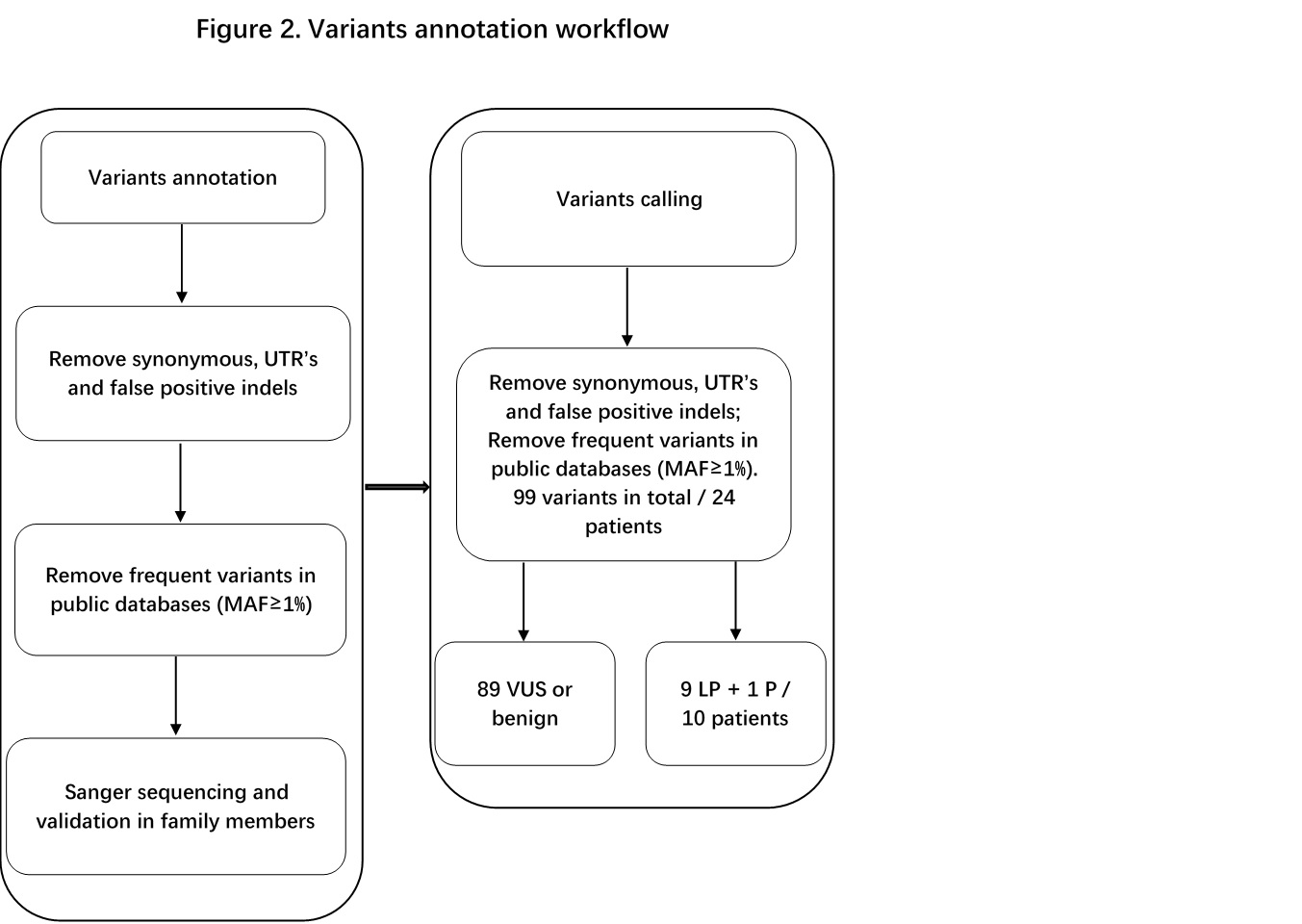

Supplement: Supplementary file 3 — Additional file 3: Figure S2. Variants annotation workflow. [file 12967_2021_2832_MOESM3_ESM.jpg]

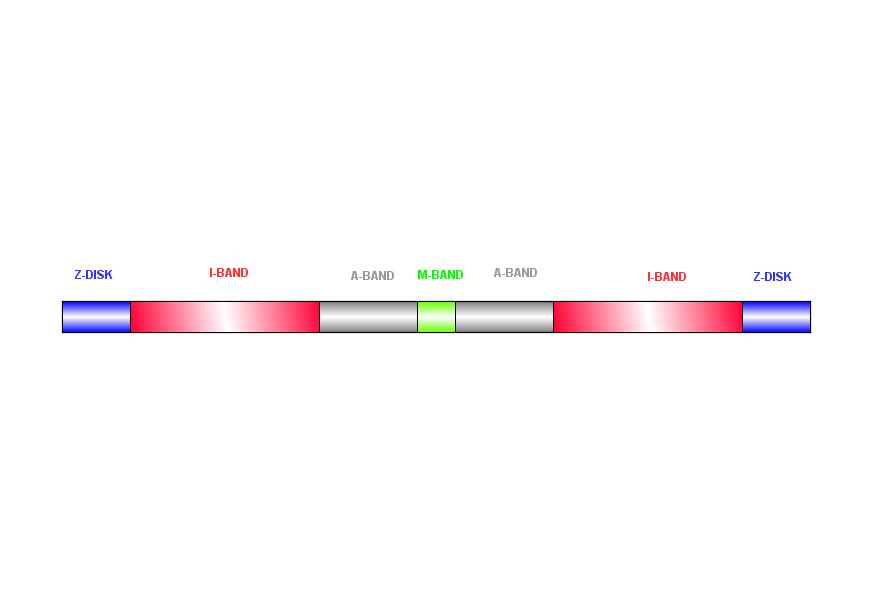

Supplement: Supplementary file 5 — Additional file 5: Figure S3. Structure and domains of TTN. [file 12967_2021_2832_MOESM5_ESM.png]

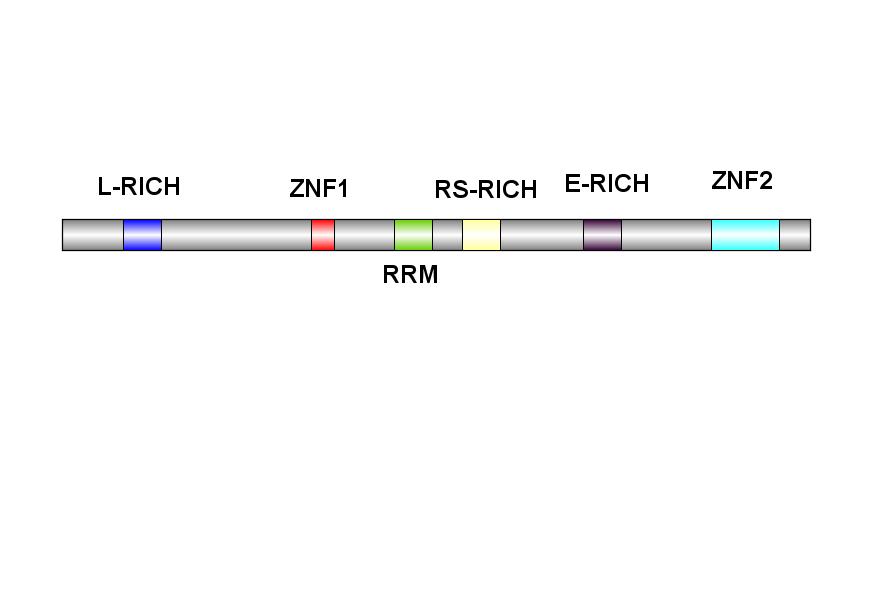

Supplement: Supplementary file 7 — Additional file 7: Figure S4. Structure and domains of RBM20. [file 12967_2021_2832_MOESM7_ESM.png]

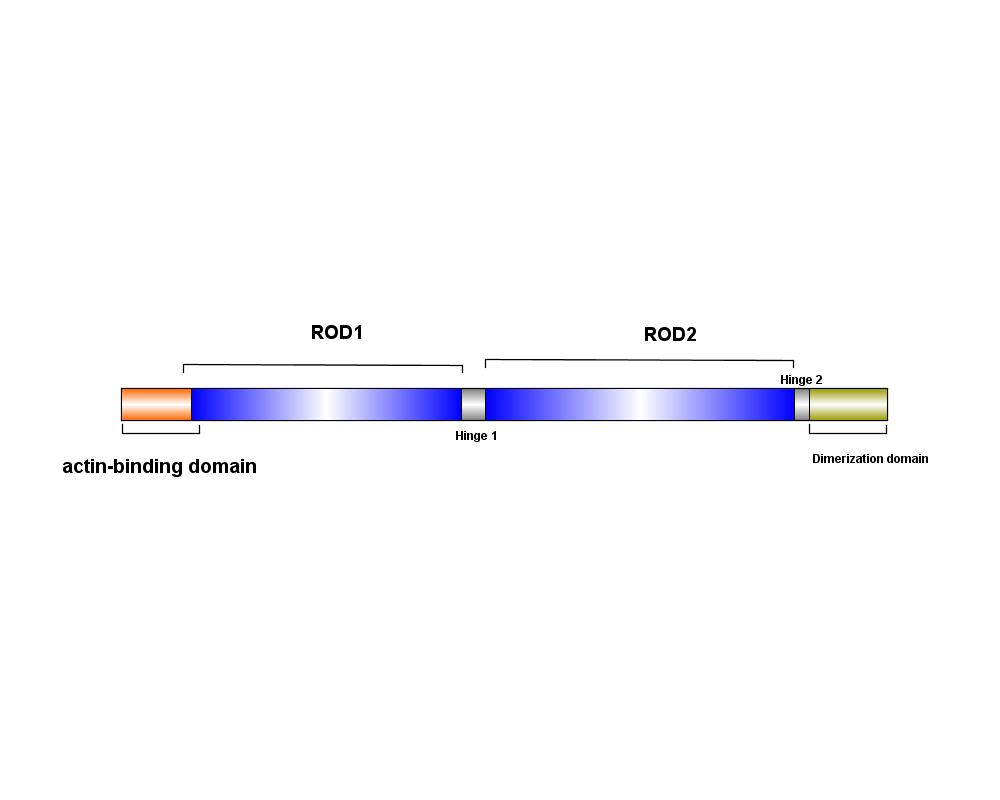

Supplement: Supplementary file 8 — Additional file 8: Figure S5. Structure and domains of FLNC. [file 12967_2021_2832_MOESM8_ESM.png]
