# Supplementary material for: Genetic analysis using targeted next-generation sequencing of sporadic Chinese patients with idiopathic dilated cardiomyopathy
Source: J Transl Med. 2021 May 3;19:189. doi: 10.1186/s12967-021-02832-3 (PMC8091742; doi:10.1186/s12967-021-02832-3)
Supplement: Supplementary file 6 — Additional file 6: Table S3. Characteristics of DCM patients, according to the presence or absence of TTNtv. [file 12967_2021_2832_MOESM6_ESM.docx]

| Characteristics | TTNtv carriers (n=7) | DCM without TTNtv (n=17) | *p* |
| --- | --- | --- | --- |
| Female gender, n(%) | 0 | 5 (29.4%) | 0.272 |
| Age at onset, years | 41.6 ± 9.1 | 39.1 ± 13.5 | 0.298 |
| Alcohol | 2 (28.6%) | 2 (11.8%) | 0.552 |
| NYHA III or IV, n(%) | 6 (85.7%) | 15 (88.2%) | 1.0 |
| Atrial fibrillation | 1 (14.3%) | 1 (5.9%) | 0.507 |
| Ventricular tachycardia | 0 | 2 (11.8%) | 1.0 |
| Complete RBBB/LBBB | 1 (14.3%) | 0 | 0.292 |
| LVEF, % | 26.6±6.8 | 27.1 ± 11.1 | 0.062 |
| LVEDD, mm | 68.4±2.7 | 70.8 ± 9.2 | 0.082 |
| ICD, n(%) | 0 | 2 (11.8%) | 1.0 |

**Table S3. Characteristics of DCM patients, according to the presence or absence of TTNtv**

*All variables are given as either number present (% of total number) or mean ± SD, except for NT-proBNP (N-terminal B-type natriuretic peptide), which is given as mean and range.*

*NYHA, New York Heart Association functional classification; ICD, implantable cardioverter defibrillator; RBBB/LBBB, right bundle branch block/left bundle branch block; LVEDD, left ventricular end-diastolic dimension;*

*Fisher’s exact test for categorical variables comparison. student’s t-test for continuous variables comparison.*
